# Ceftriaxone Efficacy for *Mycobacterium avium* Complex Lung Disease in the Hollow Fiber and Translation to Sustained Sputum Culture Conversion in Patients

**DOI:** 10.1093/infdis/jiad545

**Published:** 2023-11-30

**Authors:** Devyani Deshpande, Gesham Magombedze, Gunavanthi D Boorgula, Moti Chapagain, Shashikant Srivastava, Tawanda Gumbo

**Affiliations:** Baylor University Medical Center, Dallas; Mathematical Modeling and AI Department, Praedicare Inc, Dallas; Department of Medicine, School of Medicine, University of Texas at Tyler; Department of Cellular and Molecular Biology, School of Medicine, University of Texas Health Science Center at Tyler; Baylor University Medical Center, Dallas; Department of Medicine, School of Medicine, University of Texas at Tyler; Department of Cellular and Molecular Biology, School of Medicine, University of Texas Health Science Center at Tyler; Mathematical Modeling and AI Department, Praedicare Inc, Dallas; Hollow Fiber System and Experimental Therapeutics Laboratories, Praedicare Inc, Dallas, Texas

**Keywords:** γ slope, extinction mathematics, sustained sputum culture conversion, virtual clinical trials

## Abstract

**Background:**

Only 35.6%–50.8% of patients with *Mycobacterium avium* complex (MAC) pulmonary disease achieve sustained sputum culture conversion (SSCC) on treatment with the azithromycin-ethambutol-rifabutin standard of care (SOC). We tested the efficacy of ceftriaxone, a β-lactam with a lung-to-serum penetration ratio of 12.18-fold.

**Methods:**

We mimicked lung concentration-time profiles of 7 ceftriaxone once-daily doses for 28 days in the hollow fiber system model of intracellular MAC (HFS-MAC). Monte Carlo experiments were used for dose selection. We also compared once-daily ceftriaxone monotherapy to 3-drug SOC against 5 MAC clinical isolates in HFS-MAC using γ (kill) slopes, and translated to SSCC rates.

**Results:**

Ceftriaxone killed 1.02–3.82 log_10_ colony-forming units (CFU)/mL, at optimal dose of 2 g once-daily. Ceftriaxone killed all 5 strains below day 0 versus 2 of 5 for SOC. The median γ (95% confidence interval [CI]) was 0.49 (.47–.52) log_10_ CFU/mL/day for ceftriaxone and 0.38 (.34–.43) log_10_ CFU/mL/day for SOC. In patients, the SOC was predicted to achieve SSCC rates (CI) of 39.3% (36%–42%) at 6 months. The SOC SSCC was 50% at 8.18 (3.64–27.66) months versus 3.58 (2.20–7.23) months for ceftriaxone, shortening time to SSCC 2.35-fold.

**Conclusions:**

Ceftriaxone is a promising agent for creation of short-course chemotherapy.

## Teaser

Lung disease due to *Mycobacterium avium* complex shows response rates in only 40% of patients treated with the standard-of-care combination regimen. Ceftriaxone, an old drug, demonstrated better efficacy and is predicted to shorten therapy duration in patients 2.4-fold.

The standard of care (SOC) regimen for the treatment of *Mycobacterium avium* complex (MAC) lung disease consists of a combination of a macrolide (clarithromycin or azithromycin), ethambutol, and a rifamycin (rifabutin or rifampin) [1]. Among MAC subspecies, *Mycobacterium intracellulare* causes more severe disease and has poorer outcomes to SOC compared to *M avium* subspecies [2]. Moreover, a meta-analysis of prospective studies demonstrated, based on intention-to-treat analyses, that the sustained sputum culture conversion (SSCC) rate with the SOC was 53% (standard deviation [SD], 14%) at the 6-month timepoint and only 22% at therapy duration >1 year [3]. On average, patients received approximately 18 months of the SOC. In a separate meta-analysis, SOC achieved SSCC of only 43% (95% confidence interval [CI], 35.6%–50.8%) when only randomized controlled trials were analyzed [4]. The prolonged treatment often results in significant adverse events [5]. Therefore, there is an unmet need for antimicrobials with high efficacy and low toxicity and that could shorten therapy duration for pulmonary MAC.

The β-lactam antibiotics are potential therapies for pneumonia caused by slow-growing mycobacteria, including MAC [6–10]. The cephalosporin ceftazidime plus the β-lactamase inhibitor avibactam was shown to be potent in the intracellular MAC hollow fiber system model of pulmonary disease (HFS-MAC) [8, 11]. However, (1) the short half-life of 2.7 hours for both drugs, (2) the epithelial lining fluid (ELF)-to-plasma penetration of 52% for ceftazidime and 42% for avibactam, and (3) the fact that it is percentage of time concentration persisting above the minimum inhibitory concentration (MIC) (%T_MIC_) that optimizes β-lactam efficacy necessitate multiple and high parenteral doses each day [8, 11–13]. This imposes practical constraints on routine clinical use for a disease with a long treatment duration. Ceftriaxone is a more chemically stable cephalosporin, which we hypothesized would not require avibactam. Moreover, ceftriaxone has the pharmacokinetic (PK) advantage of a half-life of 8 hours. While the ceftriaxone lung penetration in patients with pneumonia was unknown till recently, it was known to achieve higher concentrations compared to plasma in bile (12.6-fold) and urine (10.47-fold), but was assumed to be lower in the lung [14]. The first direct measurement of ceftriaxone ELF-to-plasma ratios was recently published by Dong et al and was 12.18-fold, which is consistent with penetration in other tissues [14, 15]. On the other hand, since it is non-protein-bound concentrations that kill bacteria, there is a need to account for ceftriaxone's 80% protein binding. However, the mean protein ELF-to-plasma ratio in adults varies from 0.13 in healthy volunteers to 0.25 in patients with severe acute respiratory distress syndrome (ARDS) for total protein, versus 0.1 in healthy volunteers to 0.19 in ARDS for albumin [16, 17]. This means that ceftriaxone protein binding in ELF is expected to be negligible, and the measured total antibiotic concentrations in ELF are considered to reflect free (unbound) fractions [18].

Here, we tested ceftriaxone efficacy using a PK/pharmacodynamic (PD) design in the HFS-MAC, based on the human lung PKs [8, 15, 19]. Bacterial kill slopes in the HFS-MAC were calculated using an ordinary differential equation (ODE) we published elsewhere [20]:


$$\frac{dB}{dt}=r\;*\;B\;\left(1-\frac{B}{K_{max}}\right)-\gamma \;*\;B$$

where *r* is bacterial growth rates in nontreated HFS-MAC units, *B* is bacterial burden (initial bacterial burden is *B*_0_) in log_10_ colony-forming units (CFU)/mL, K_max_ is carrying capacity, and γ is the kill slope (log_10_ CFU/mL/day). From this, the time to extinction (τ) of the entire bacterial population (ie, relapse-free cure) can be calculated. These same parameters have been derived based on repetitive sampling in patients’ sputa on treatment with the SOC for pulmonary MAC and in the HFS-MAC, allowing direct translation from the HFS-MAC to patients’ SSCC in virtual clinical trials [20, 21].

## METHODS

### Bacterial Strains and Supplies

The experiments were performed using the MAC standard laboratory strain (ATCC number 700898), that is, *M avium* subspecies *hominissuis*, and a library of 30 clinical MAC isolates. Stock and expansion of bacterial cultures were performed as described previously [22–26]. THP-1 monocytes (ATCC TIB-202) were cultured in RPMI 1640 medium supplemented with 10% heat-inactivated fetal bovine serum. Ceftriaxone, azithromycin, ethambutol, and rifabutin were purchased from the Baylor University Medical Center pharmacy. Cellulosic hollow fiber cartridges were procured from FiberCell (Frederick, Maryland). BACTEC MGIT 960 mycobacterial growth tube indicator system (MGIT) and MGIT tubes were supplied from Becton Dickinson (Franklin Lakes, New Jersey).

### MICs and Intracellular Static Concentration-Response Study

We used the standard broth microdilution in cation-adjusted Mueller–Hinton broth to determine the ceftriaxone MICs of MAC, described in detail in the Supplementary Methods [27]. For the concentration versus response study in extracellular and intracellular MAC infection, we performed experiments that are described in detail in the Supplementary Methods.

### Ceftriaxone Exposure-Effect Study in the HFS-MAC

The HFS-MAC has been described extensively elsewhere, and in detail in the Supplementary Methods [8, 9, 22, 23, 28, 29]. We performed a ceftriaxone exposure-response study in the HFS-MAC, at a ceftriaxone 8-hour half-life [14, 19, 30, 31]. Since %T_MIC_ is associated with the efficacy of cephalosporins, the drug dose selection was to achieve the %T_MIC_ ranging from 0% (nontreated control) to 100%. The central compartment of each HFS-MAC unit was sampled at 0, 1, 4, 6, 7, 13, 21, and 23.5 hours after the last dose to determine the concentration-time profile for each ceftriaxone exposure. Simultaneously, the infected macrophages in the peripheral compartment of the HFS-MAC were also collected to measure the intracellular ceftriaxone concentration. For enumeration of the intracellular bacterial burden, sampling of the peripheral compartment of each HFS-MAC was performed on days 0, 2, 7, 14, 21, and 28 of treatment. After cell lysis, the samples were serially diluted and cultured on agar to estimate bacterial burden, as described above. The relationship between ceftriaxone %T_MIC_ versus intracellular MAC burden was analyzed using the inhibitory sigmoid maximal kill (E_max_) model, and the exposure mediating 80% of the E_max_ or EC_80_, calculated. We compared the HFS-MAC co-treated with avibactam for both E_max_ and the exposure mediating 50% of E_max_ (EC_50_) to determine if avibactam added any effect.

### Ceftriaxone Monotherapy Versus SOC Against 5 Clinical Isolates in the HFS-MAC

Five clinical MAC strains (2 *M avium*, 3 *M intracellulare*) were chosen because the rate of response to the SOC in the HFS-MAC reflects the 43%–53% SCC encountered in clinical trials [3, 4]. The first objective of this proof-of-concept experiment was to generalize the effect of ceftriaxone EC_80_ exposure identified in the exposure-effect HFS-MAC with *M. avium* subspecies *hominissuis*, by examining its effect in 5 MAC clinical strains. The second objective was to compare the microbial kill of ceftriaxone monotherapy to that of the 3-drug SOC (azithromycin-ethambutol-rifabutin) at concentration-time profiles achieved in the lungs with the recommended clinical doses of each drug [32–34]. Bacterial burden (log_10_ CFU/mL) trajectories were analyzed using γ-slopes and time to extinction as described elsewhere and in equation 1 [20]. These results were then translated to sputum (τ) using nonlinear translation factors.

### PK Analyses

Ceftriaxone, azithromycin, ethambutol, and rifabutin concentrations in the HFS-MAC were analyzed by liquid chromatography with tandem mass spectrometry assays, as described elsewhere [8–10], and in detail in the Supplementary Methods. Measured drug concentrations were modeled using ADAPT 5 software [35]. We examined 1- and 2-compartment models, and the best model was selected using the Akaike information criterion [36].

### Ceftriaxone Clinical Dose Selection In Silico Experiments

We performed Monte Carlo experiments to identify the dose of intravenous ceftriaxone that would achieve the EC_80_ in the lungs of 10 000 virtual patients with pulmonary MAC, assuming an ELF-to-plasma ratio of 12.18 [15]. The population PK parameters of Fraschini et al were used in steps described in the Supplementary Methods [19, 37].

### Model Parameter Estimation

The ODE model, equation 1 was fit to HFS-MAC data for (1) each individual MAC strain to determine their respective growth rates (*r*) and carrying capacity (K_max_) when there is no treatment, and (2) all strains pooled together to determine a growth rate that summarizes all strains. Then, these parameters we used in the model, but fixed, as the model was fit to SOC HFS treatment data and the ceftriaxone HFS-MAC treatment data to estimate the kill rates (γ) for each strain and all strains combined. The model was fitted to the data under a Gaussian likelihood using a Markov chain Monte Carlo (MCMC) algorithm implemented in R software (FME package). Parameter posterior distributions were drawn from 15 000 MCMC samples discarding a burn-in period assuming uniform priors for all fitted parameters. Chains were analyzed visually and using the Coda R package for convergence. The 95% credible intervals (CrIs) for the estimated parameters were computed using the 2.5th–97.5th quantiles of the MCMC algorithm.

## RESULTS

### Ceftriaxone MIC Distribution and Static Concentrations Versus Effect

**Figure 1.**
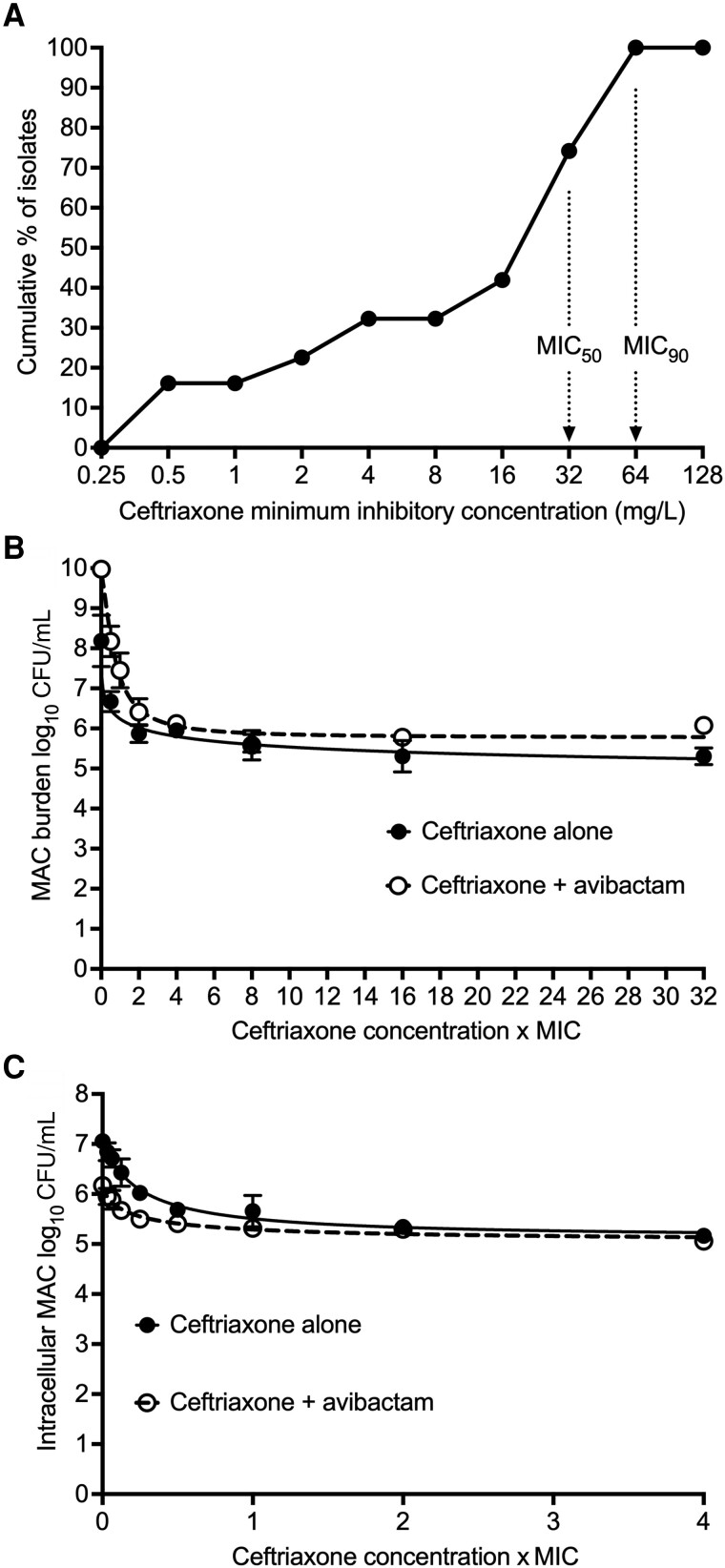
Ceftriaxone minimum inhibitory concentration and of static concentrations. *A*, Cumulative minimum inhibitory concentrations (MICs) of 30 clinical isolates, showing the MIC_50_ and MIC_90_. The modal MIC was 32 mg/L. *B* and *C*, Symbols are mean bacterial burden (n = 3) and error bars are standard deviation, after 7 days of drug co-incubation. *B*, Extracellular *Mycobacterium avium* complex (MAC). *C*, Intracellular MAC. Abbreviations: CFU, colony-forming units; MAC, *Mycobacterium avium* complex; MIC, minimum inhibitory concentration.

The MIC of MAC laboratory isolate (ATCC number 700898) was 4 mg/L. Figure 1*A* shows the MIC distribution in 30 clinical isolates. The lowest ceftriaxone MIC at which 50% (MIC_50_) and 90% (MIC_90_) of isolates were inhibited was 32 mg/L and 64 mg/L, respectively. The tentative epidemiological cutoff value was therefore an MIC of 64 mg/L.

Figure 1 *B* and 1*C* shows the results of the static concentration-response studies, in extracellular and intracellular MAC. First, microbial effect maxed out between 1 and 4 times MIC in all instances, consistent with time-driven efficacy for cephalosporins and other β-lactams [13]. Second, we tested the null hypothesis that EC_50_ and E_max_ were the same with and without avibactam. Figure 1*B* shows the extracellular MAC results. The EC_50_ with and without avibactam (95% CI) was 0.67 (.51–.84) times MIC versus 0.58 (.26–1.42) times MIC, respectively, while the E_max_ was 4.23 (3.74–4.71) versus 3.23 (1.88–4.58) log_10_ CFU/mL, respectively (*P* = .28). Figure 1*C* shows the intracellular MAC results. The EC_50_ with and without avibactam was 0.20 (.03–.26) times MIC and 0.25 (.12–.37) times MIC, respectively, while the E_max_ was 1.16 (.84–1.47) and 1.96 (1.55–2.38) log_10_ CFU/mL, respectively (*P* = .006). Since the EC_50_ for intracellular MAC was better than that for extracellular MAC (<0.0001), we further hypothesized that this could be due to higher intracellular ceftriaxone concentrations compared to extracellular concentrations.

### Ceftriaxone Exposure-Effect HFS-MAC Study With and Without Avibactam

**Figure 2.**
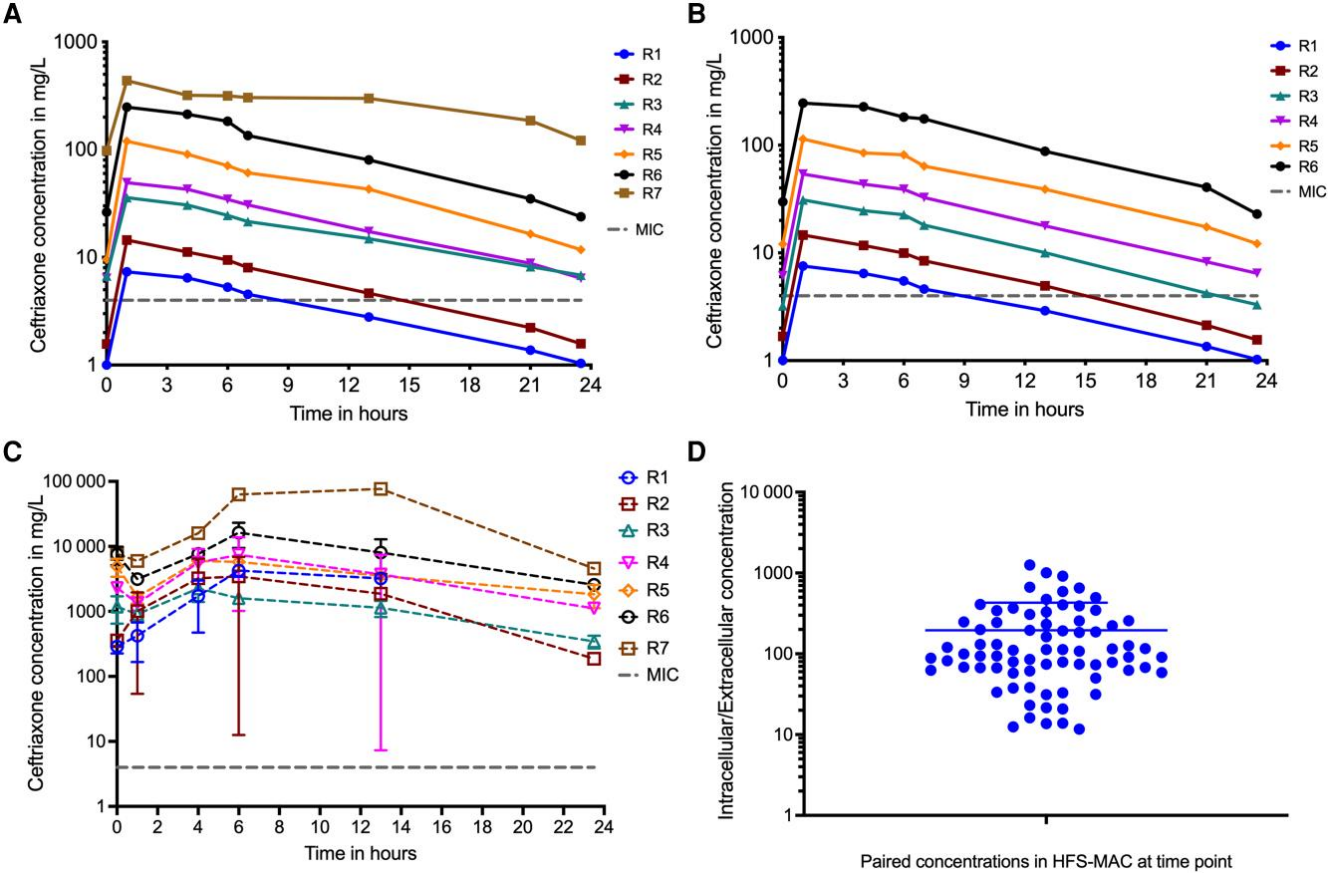
Ceftriaxone pharmacokinetics and time-kill curves in the hollow fiber system model of intracellular *Mycobacterium avium* complex (HFS-MAC). R1, R2, R3, R4, R5, R6, and R7 are the corresponding regimens in each panel. The abscissa is on log_10_ scale for all. *A*, Concentration-time profiles achieved by each of the 7 ceftriaxone doses administered without avibactam examined in the HFS-MAC, with a log_10_ x-axis scale. *B*, Concentration-time profiles achieved by each of the 6 doses administered with avibactam in the HFS-MAC with a log_10_ x-axis scale. *C*, Intracellular concentrations of ceftriaxone were so high that the abscissa maxima was 100 000 mg/L compared to 1000 mg/L for extracellular concentrations in (*A*) and (*B*). *D*, Ratio of the intracellular to extracellular concentration in HFS-MAC samples at all time points, at steady state. Abbreviations: HFS-MAC, hollow fiber system model of intracellular *Mycobacterium avium* complex; MIC, minimum inhibitory concentration.

In the HFS-MAC, measured avibactam concentrations were as shown in Supplementary Figure 1. Ceftriaxone concentration-time profiles achieved on day 28 of study were as shown in Figure 2*A* for the ceftriaxone alone, and Figure 2*B* for ceftriaxone plus avibactam–treated HFS-MAC units. Figure 2*A* and 2*B* also shows the ceftriaxone %T_MIC_ achieved by each dose, based on the measured drug concentrations. ADAPT PK model parameter estimates were a ceftriaxone clearance of 0.03 (SD, 0.005) L/hour, volume of 0.293 (SD, 0.026) L, and a half-life of 7.66 (SD, 1.55) hours in the HFS-MAC. The observed versus model-predicted concentrations were a straight line with a slope of 1.02 (95% CI, .99–1.05), indicating no bias (*r*^2^ = 0.98). We also measured intracellular ceftriaxone concentrations and infected THP-1 monocyte volumes on day 28, with results of all HFS-MAC units combined and shown in Figure 2*C*. The intracellular ceftriaxone concentration decline was slower than for extracellular, hence a longer intracellular half-life. Figure 2*D* shows the ratio of intracellular-to-extracellular ceftriaxone concentration for each sampling time point in each HFS-MAC unit; the median intracellular-to-extracellular ratio was 103.6.

**Figure 3.**
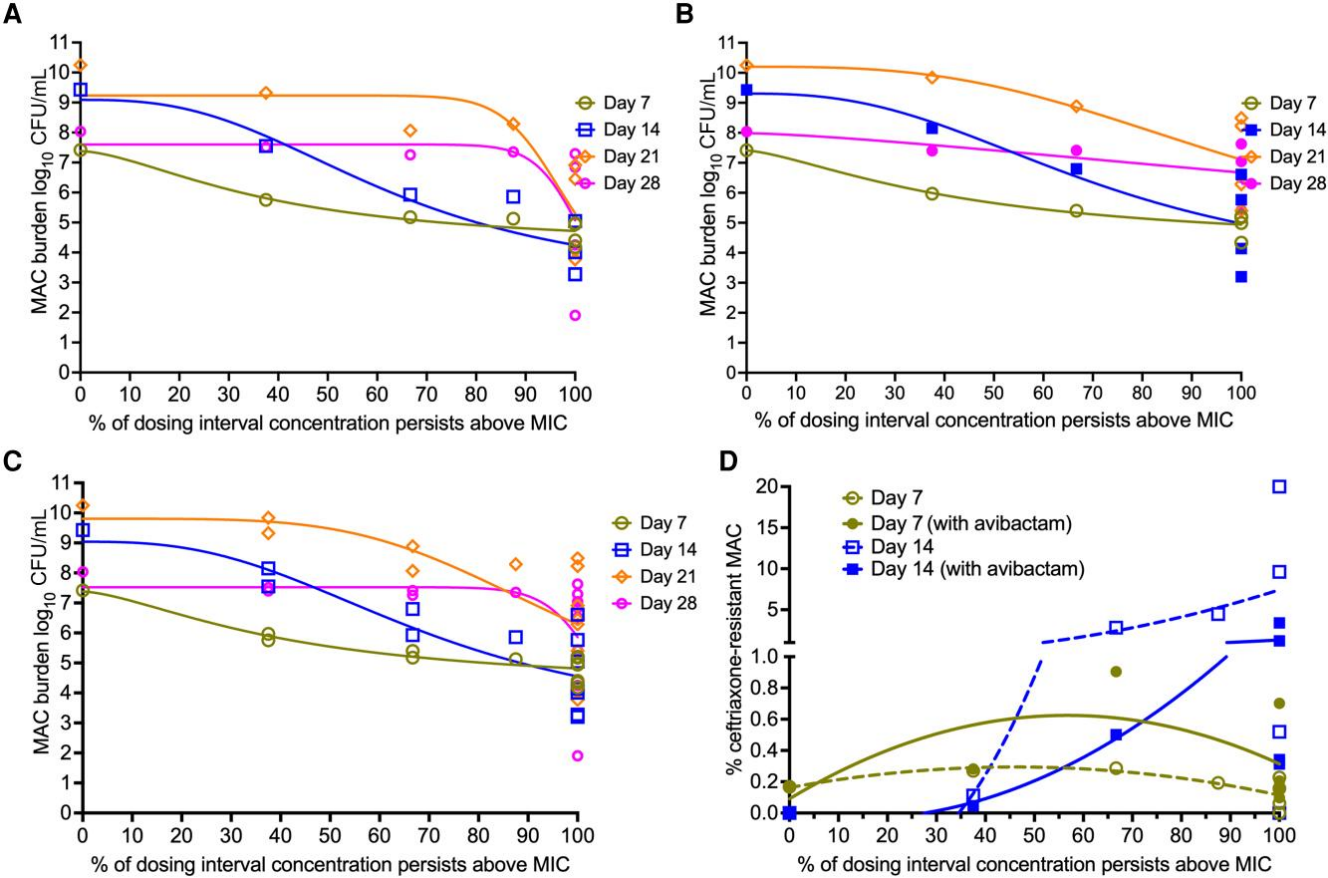
Pharmacokinetics/pharmacodynamics for microbial kill and acquired drug resistance in the hollow fiber system model of intracellular *Mycobacterium avium* complex (HFS-MAC). Inhibitory sigmoid maximal kill models for days 7 to 28 for ceftriaxone alone (*A*), ceftriaxone with avibactam (*B*), and all HFS-MAC replicates combined (*C*). Day 2 regressions failed to achieve convergence and are not shown. *A–C*, In all analyses, it can be seen that maximum effect was at around 100% of the dosing interval. *D*, The relationship between ceftriaxone-resistant MAC colony-forming units (CFU)/mL as a percentage of the total CFU/mL in each HFS-MAC was analyzed using the antibiotic resistance arrow of time model [28]. Avibactam protected against ceftriaxone resistance, with apogee (ie, maximum resistance %) ratios of non-avibactam- to avibactam-containing regimens of 2.0 on day 7 and 8.02 on day 14, which means that avibactam reduced the proportion of ceftriaxone-resistant bacteria by 2–8.02 times. Abbreviations: CFU, colony-forming units; MAC, *Mycobacterium avium* complex; MIC, minimum inhibitory concentration.

Figure 3 *A* shows the inhibitory sigmoid E_max_ for %T_MIC_ versus bacterial burden in HFS-MAC treated with ceftriaxone without avibactam, while Figure 3*B* shows those treated with both ceftriaxone plus avibactam. Supplementary Table 1 shows results of null hypothesis testing, and demonstrates that avibactam does not change the EC_50_ or E_max_ for each sampling day. The null hypothesis was not rejected. Mathematically, this means that all of the data for HFS-MAC with or without avibactam can be combined in a single regression, improving the number of datapoints and parameter estimates. Figure 3*C* shows inhibitory sigmoid E_max_ models with combined datasets. Parameter estimates changed between sampling days, as seen with other drugs [20]. The day with the best Akaike information criteria scores was day 7. On day 7, the relationship between bacterial burden and ceftriaxone %T_MIC_ was described by the equation:


$$\begin{array}{rl} \mathrm{Effect}(\mathrm{lo}g_{10}\;\mathrm{CFU}/\mathrm{mL})= & \;[{7.38-3.23}^{*}\text{\%}{T_{\mathrm{MIC}}}^{1.55}]\\ & /[{41.86^{1.55}+\text{\%}T_{\mathrm{MIC}}}^{1.55}]\;r^{2}=0.86 \end{array}$$

where the EC_50_ was %T_MIC_ of 41.86%, E_con_ was 7.38 log_10_ CFU/mL, E_max_ was 3.23 log_10_ CFU/mL, and Hill factor was 1.55. This translates to an EC_80_ %T_MIC_ of 100%. The maximal kill was 3.10 log_10_ CFU/mL below day 0 (stasis) on day 28.

The relationship between ceftriaxone %T_MIC_ versus ceftriaxone-resistant MAC log_10_ CFU/mL is shown in Figure 3*D*; resistance was defined as growth on agar supplemented with 4 times ceftriaxone MIC. There was no antimicrobial resistance (AMR) on day 2, but AMR was encountered by day 7 and day 14, and the relationship followed the “antibiotic resistance arrow of time” model, which suggests that ceftriaxone is a bona fide anti-MAC agent just like macrolides (*r*^2^ = 0.54) [28]. On the other hand, avibactam did not lower the %T_MIC_ required to completely suppress resistance.

### Ceftriaxone Versus SOC Against 5 Strains in the HFS-MAC

The concentration-time profiles of ceftriaxone and SOC drugs that were measured for the duplicate HFS-MAC per condition per bacterial strain were as shown in Supplementary Figure 2*A*. MICs are also shown in Supplementary Table 2. The between HFS-MAC unit %CV was <10% at all time points for all drugs, satisfying quality control criteria [38]. The ceftriaxone 0-24 hour area under the concentration-time curve (AUC_0–24_) achieved in the HFS-MAC was 15.01 ± 0.49 mg*hour/L (AUC_0–24_/MIC = 120.08) for azithromycin, 14.14 ± 0.50 mg*hour/L (AUC_0–24_/MIC = 7.07) for ethambutol, and 5.29 ± 0.39 mg*hour/L (AUC_0–24_/MIC = 42.32) for rifabutin, in the range identified in lung lesions and ELF [32–34]. Ceftriaxone achieved %T_MIC_ of 100% in all HFS-MAC. The ceftriaxone ADAPT PK model output was a clearance of 0.014 ± 0.0 L/hour, a volume of 0.178 ± 0.09 L, and a half-life of 8.73 ± 0.68 hours in the HFS-MAC, with a PK model predicted versus observed concentrations with an *r*^2^ = 0.99.

Time-kill curves with ceftriaxone monotherapy and SOC are shown in Supplementary Figure 2*B–F*. The SOC achieved a partial response in 1 strain and then failed (Supplementary Figure 2*B*) and killed 2 strains effectively (Supplementary Figure 2*C* and 2*D*), but completely failed in 2 strains (Supplementary Figure 2*E* and 2*F*) [3]. Ceftriaxone killed all 5 strains below day 0, with maximal kill varying from 1.02 to 3.82 log_10_ CFU/mL.

**Figure 4.**
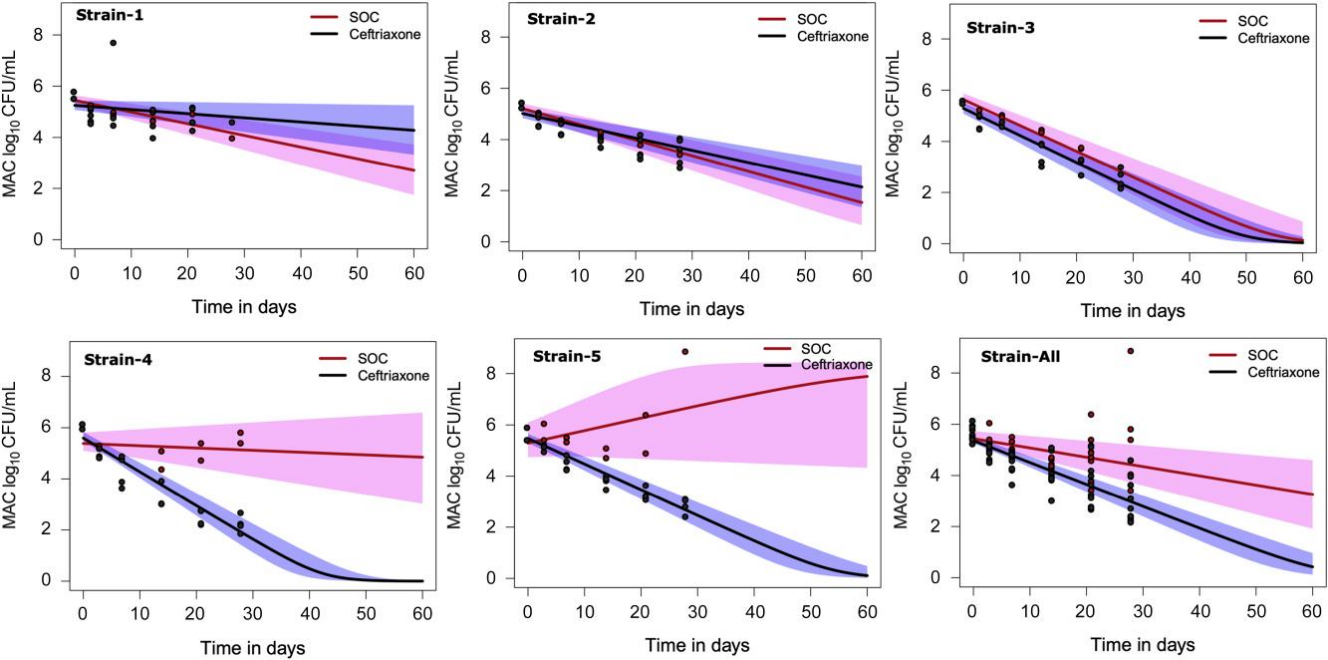
Comparison of bacterial trajectories under treatment with standard of care (SOC) or ceftriaxone. Observed bacterial burden values are shown as circles, and model fitting as lines, with 95% confidence intervals (CIs). The pink depicts SOC while periwinkle depicts ceftriaxone monotherapy. In strains 1, 2, and 3, the 95% CIs overlap, which means ceftriaxone performed as well as SOC. In strains 4 and 5, ceftriaxone performed well while SOC failed. When all strains were combined and the vectors added, ceftriaxone monotherapy performed better than SOC, with a steeper γ. Abbreviations: CFU, colony-forming units; MAC, *Mycobacterium avium* complex; MIC, minimum inhibitory concentration.

Next, all data (CFU/mL and time to positivity [TTP]) were modeled using our ODE, and yielded trajectories shown in Figure 4 and parameter estimates shown in Table 1. The parameter estimates allow a quantitative description of between-strain variation. In Table 1, Figure 4, and Supplementary Figure 3 (growth rates), *B*_0_, reflecting infectivity, varied 5.50-fold (549.5%) between the strains while the growth rate (*r*) varied statistically from strain to strain, consistent with the heterogeneity encountered in patients. The heterogeneity of SOC efficacy is demonstrated in Table 1 and Figure 4 trajectories by considering the lowest lower CI of γ of 0.03 log_10_ CFU/mL/day versus the highest of 0.57 log_10_ CFU/mL/day (19-fold range). This means that SOC γ varied from virtually no effect to high speeds of microbial kill. On the other hand, ceftriaxone γ demonstrated less heterogeneity, ranging from 0.27 log_10_ CFU/mL/day versus the highest of 0.58 log_10_ CFU/mL/day (2-fold range). Table 1 and Figure 4*F* further demonstrate that the median γ for all 5 strains for ceftriaxone was statistically higher than that for the SOC.

**Table 1.** Parameter Estimates for Ordinary Differential Equations in a Hollow Fiber System Model of Intracellular *Mycobacterium avium* Complex in All Isolates

	Baseline Inoculum	Growth Rate	SOC γ	Ceftriaxone γ
MAC Isolate	Log_10_ CFU/mL (95% CI)	Log_10_ CFU/mL/d (95% CI)	Log_10_ CFU/mL/d (95% CI)	Log_10_ CFU/mL/d (95% CI)
1	5.58 (5.53–5.72)	0.26 (.22–.30)	0.37 (.33–.40)	0.30 (.27–.33)
2	5.30 (5.26–5.42)	0.35 (.31–.39)	0.49 (.46–.52)	0.46 (.44–.49)
3	5.53 (5.50–5.60)	0.30 (.26–.33)	0.53 (.49–.57)	0.54 (.51–.58)
4	6.04 (5.98–6.15)	0.22 (.18–.26)	0.24 (.18–.30)	0.52 (.49–.56)
5	5.71 (5.47–5.90)	0.25 (.20–.32)	0.14 (.03–.27)	0.48 (.45–.51)
All	5.51 (5.38–5.66)	0.30 (.27–.33)	0.38 (.34–.43)	0.49 (.47–.52)

Abbreviations: CFU, colony-forming units; CI, confidence interval; MAC, *Mycobacterium avium* complex; SOC, standard of care.

### Translation to Sustained Sputum Culture Conversion in Patients

**Figure 5.**
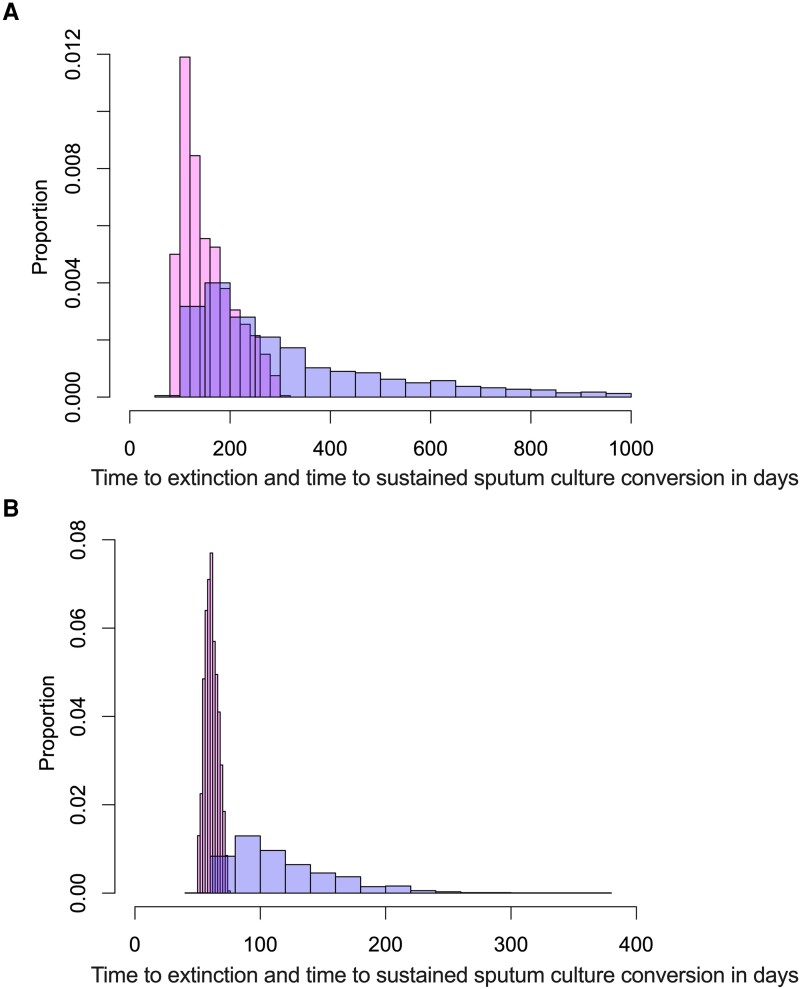
Translation from the hollow fiber system model of intracellular *Mycobacterium avium* complex (HFS-MAC) to sustained sputum culture conversion (SSCC) rates. Shown are histograms of proportions versus time to bacterial population extinction in the HFS-MAC (pink) translated to sputum SSCC rates (periwinkle) in 1000 patients. *A*, Standard of care (SOC) translation. Time to extinction in the HFS has a median value of 139 days; however, in patients, it takes a median of 249 days with a long tail that stretches to beyond 800 days with an asymmetric distribution. *B*, Data for ceftriaxone monotherapy; the x-axis scale stretches only to 400 days for ceftriaxone versus 1000 days for SOC. It took 61 days to extinction in the HFS-MAC, which translated to median time to SSCC rates of 106 days in sputum of patients, with a narrower distribution.

Elsewhere in the past, serial sputum CFU/mL readouts (calculated from TTP) from patients with MAC lung disease treated on SOC (n = 30) were used to determine the time it takes to clear MAC bacteria by applying the developed and parameterized mechanistic model by simulating MAC depletion until bacteria are <0.01 CFU/mL. A clinical MAC treatment in silico simulation with bacteria baseline variability, bacteria growth rate, and derived bacteria kill rates was then generated for a 1000-patient virtual population. Applying the developed mechanistic model with data from the HFS-MAC, a mapping translation was performed following the approach of Magombedze et al [20, 21]. Figure 5 shows the application of translation of the ceftriaxone regimen from the HFS-MAC to reflect how this will perform in a real-world MAC treatment clinical study. The SOC SSCC rates at 6 months (180 days), using the MGIT cutoff of 44 days, translated to 39.3% (36%–42%), which is similar to the SSCC of 43% (95% CI, 35.6%–50.8%) in meta-analyses of randomized controlled clinical trials [4]. This is an important validation that our modeling predictions match with the clinical trial findings. The SOC achieved SSCC rates of 50% at 8.18 (95% CI, 3.64–27.66) months versus 3.58 (95% CI, 2.20–7.23) months for ceftriaxone monotherapy.

### Translation to Optimal Ceftriaxone Dose Selection for Use in Patients

**Figure 6.**
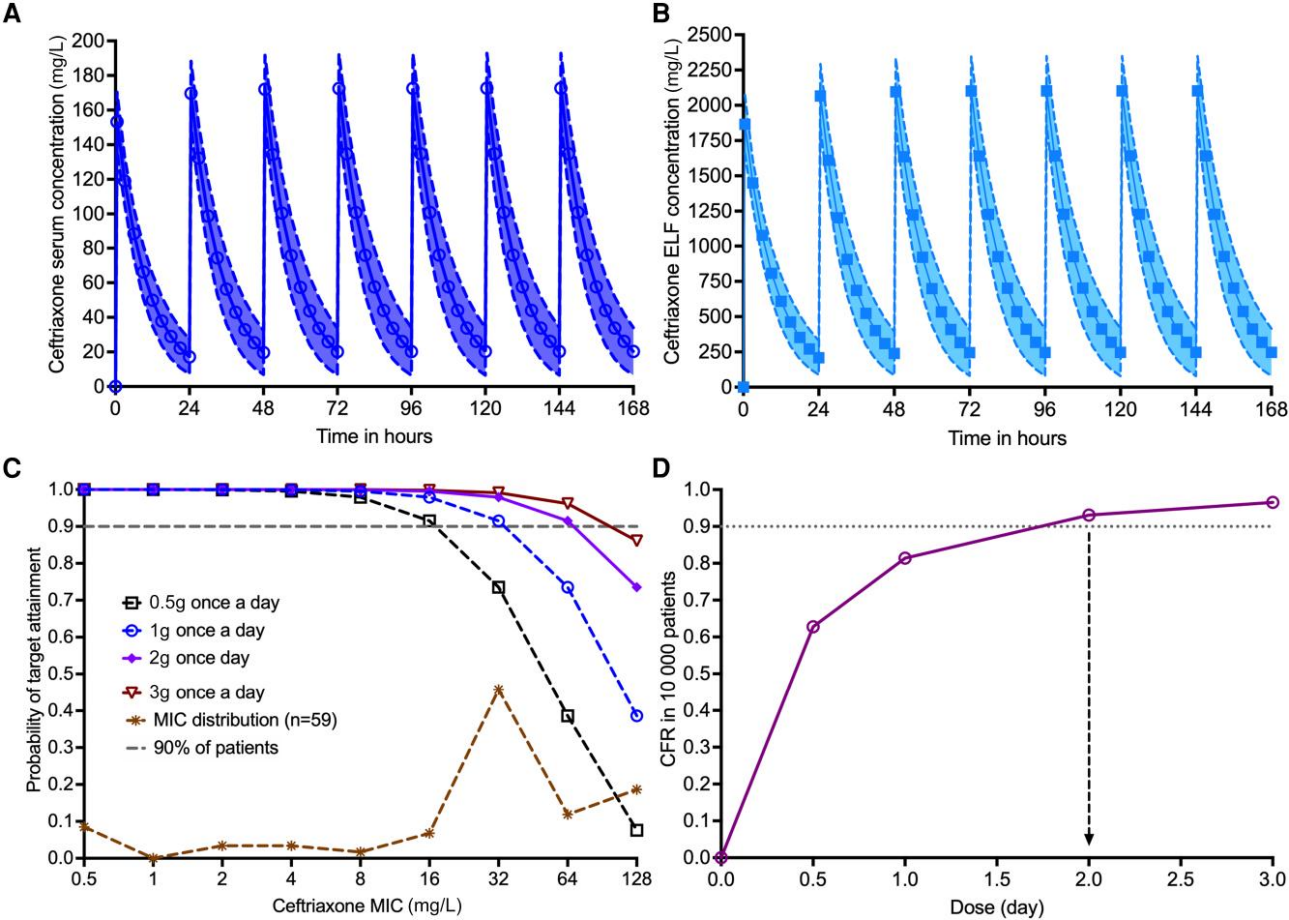
Probability of optimal ceftriaxone exposure target attainment. The target EC_80_ is the percentage of time concentration persisting above the minimum inhibitory concentration (MIC) of 100%. *A*, Simulated ceftriaxone serum concentrations reach steady state by 120 hours. *B*, Simulated ceftriaxone epithelial lining fluid (ELF) concentrations rare shown using a different scale of the y-axis because of the range of concentrations. *C*, Probability of target attainment (PTA) in 100 000 virtual patients given ceftriaxone pharmacokinetic variability and penetration into lung lesions. Cumulative MICs in 59 *Mycobacterium avium* complex isolates show the range of MICs likely to be encountered in the clinic. *D*, The cumulative faction of response (CFR) of >90% was achieved by a ceftriaxone intravenous dose of 2 g administered once daily. At this dose, as shown in (*C*), the PTA falls below 90% at an MIC of 64 mg/L, which is the pharmacokinetic/pharmacodynamic susceptibility breakpoint.

Figure 6 shows the probability of target attainment (PTA) against a range of MICs in 59 clinical isolates (30 from this study plus 29 from the literature [39]) when ceftriaxone is administered once daily, for achievement of EC_80_ %T_MIC_ of 100% in the lung. The PTA of 0.5 g, 1 g, 2 g, and 3 g daily dose fell below 90% at MICs of 16, 32, 64, and 128 mg/L, respectively. The cumulative fraction of response was 93.07% for the dose of 2 g/day, making this the optimal dose. With this dose, the PK/PD-derived susceptibility breakpoint was 64 mg/L.

## DISCUSSION

First, ceftriaxone was potent against both extracellular and intracellular MAC. However, the potency (EC_50_) was better against intracellular MAC than extracellular MAC, due to the high intracellular-to-extracellular concentration ratio, which in the HFS-MAC was 10-fold higher than that observed with ceftazidime in the past [7]. This PK aspect is crucial to treatment of pulmonary MAC, for which the lair of MAC is intracellular in monocyte-lineage cells in granulomatous and necrotic lesions [40, 41]. Moreover, ceftazidime-avibactam has a serum to lung ELF penetration ratio of only 0.32 compared with 12.8 for ceftriaxone [14, 15, 42]. Based on the studies described here, we identified the ceftriaxone dose required to attain maximal microbial kill using Monte Carlo experiments as a 2 g once-daily dose, with a PK/PD susceptibility breakpoint MIC of 64 mg/L. The dose of 2 g/day for several months is well tolerated by patients and is routinely administered for such diseases such as osteomyelitis [43, 44].

Second, avibactam did not improve ceftriaxone potency (EC_50_) or efficacy (measured as E_max_) in the HFS-MAC. This is to be expected given that MAC has not been demonstrated to carry BlaC. This β-lactamase independent efficacy is an advantage over other cephalosporins such as ceftazidime-avibactam [8]. While avibactam protected against ceftriaxone resistance by lowering the proportion of ceftriaxone-resistant CFU/mL at each exposure by a factor of 2.0–8.02 times, it did not decrease the %T_MIC_ that ceftriaxone completely suppresses AMR. Thus, avibactam will have no practical effect on optimal ceftriaxone dosing. Moreover, ceftriaxone will be administered in combination with other bactericidal drugs for treatment of pulmonary MAC; thus, ceftriaxone resistance will likely be abrogated by companion drugs.

Third, ceftriaxone exhibited considerable efficacy against several MAC strains in the HFS-MAC. In some strains, ceftriaxone killed up to 10^4^ CFU/mL from baseline, better than all other drugs tested in the system (such as macrolides, ethambutol, and rifamycins), except for omadacycline, which killed >10^5^ CFU/mL from baseline [20]. This is crucial considering that in patient lesions the MAC median burden is 1.5 × 10^5^ (range, 1.7 × 10^4^–1.6 × 10^6^) CFU/mL in cavitary lesions [40, 41]. Unlike the SOC, which killed only 2 of 5 clinical MAC isolates, ceftriaxone was effective against all strains [3]. Use of γ makes the comparison more succinct: The SOC γ ranged had a 19-fold range (extensive heterogeneity) versus the 2-fold range for ceftriaxone. This suggests that ceftriaxone will likely be effective across many clinical strains with minimal heterogeneity.

Fourth, one of our main findings is that ceftriaxone could lead to a shorter course of chemotherapy for MAC lung disease. That is because of a faster γ for ceftriaxone compared to the SOC; the predicted time to complete clearance of all lung bacteria in patients in the virtual clinical trial was shorter for ceftriaxone than for the SOC. This significant reduction in time to clear MAC translated to reduction in time to SSCC and hence minimal duration of therapy for relapse-free cure by a median of 2.35-fold (95% CI, 1.66- to 3.8-fold) for ceftriaxone compared to SOC. Our results demonstrate the potential of the ceftriaxone treatment to reduce duration of MAC treatment on novel combination regimens, perhaps with newer antibiotics such as omadacycline, epetraborole, and SPR720 [45–47]. Alternatively, on both safety and efficacy grounds, ceftriaxone would be a good replacement the nonoral drugs such as amikacin [1, 48].

There are some limitations to our study. First, the PK/PD limitation is that we did not perform a formal dose fractionation study. It could be that the unlike with gram-positive cocci and gram-negative bacilli, ceftriaxone is not a %T_MIC_ linked drug against MAC [13]. Second, the estimates for ELF-to-plasma ratios come from a single clinical study and had been assumed to be lower than what was demonstrated [14, 15]. Thus, caution is required in interpreting our results. Third, even though ceftriaxone treatment will be once daily, the intravenous route still poses a challenge for a disease currently treated with oral regimens. Fourth, our results will need to be confirmed in the clinic. Finally, our results were generated using ceftriaxone monotherapy, and its effectiveness in combination therapy is as of yet to be shown. In that respect, drugs with which ceftriaxone does not demonstrate antagonism will need to be identified in further HFS-MAC studies [9].

In summary, ceftriaxone has a potent bactericidal effect against multiple MAC strains. We propose to combine ceftriaxone with other effective anti-MAC agents to develop new and shorter duration chemotherapy regimens to treat pulmonary MAC infections.

## Supplementary Data

Supplementary materials are available at *The Journal of Infectious Diseases* online (http://jid.oxfordjournals.org/). Supplementary materials consist of data provided by the author that are published to benefit the reader. The posted materials are not copyedited. The contents of all supplementary data are the sole responsibility of the authors. Questions or messages regarding errors should be addressed to the author.

## Supplementary Material

jiad545_Supplementary_Data

## Notes

***Author contributions.*** All named authors meet the ICMJE criteria for authorship for this article, take responsibility for the integrity of the work, and have given their approval for this version to be published. Conceptualization and design: D. D. and T. G. MIC determination experiments: D. D., G. D. B., and S. S. HFS-MAC experiments: D. D., S. S., M. C., and T. G. PK/PD modeling and mathematical simulations: T. G. Ordinary differential equation implementation, extinction mathematics, and translation to sustained sputum culture conversion in patients: G. M. All authors reviewed and approved the final version of the manuscript.

***Data availability.*** The raw data for the results presented in the manuscript are available from the corresponding author upon reasonable request.

***Financial support*.** TG received Open Access funding from Praedicare Inc. D. D. received funding for this work from the Thoracic Foundation (Boston, Massachusetts). S. S. received funding from the Eunice Kennedy Shriver National Institute of Child Health and Human Development (grant number 1R01HD099756), the National Institute of Allergy and Infectious Diseases (grant number 1R21AI148096), and the American Thoracic Foundation/Insmed Research Award in Non-Tuberculous Mycobacteria Lung Disease. T. G. received funding for this study from the National Institute of Allergy and Infectious Diseases (grant number R56 AI111985).
