# Supplementary material for: Ceftriaxone Efficacy for Mycobacterium avium Complex Lung Disease in the Hollow Fiber and Translation to Sustained Sputum Culture Conversion in Patients
Source: J Infect Dis. 2023 Nov 30;230(2):e230–40. doi: 10.1093/infdis/jiad545 (PMC11326821; doi:10.1093/infdis/jiad545)
Supplement: jiad545_Supplementary_Data [file jiad545_supplementary_data.docx]

**Ceftriaxone efficacy for *Mycobacterium avium complex* lung disease in the hollow fiber and translation to sustained sputum culture conversion in patients.**

Devyani Deshpande, Gesham Magombedze, Gunavanthi D. Boorgula, Moti Chapagain, Shashikant Srivastava, Tawanda Gumbo

**Supplementary Figures**

Supplementary Figure S1. Avibactam concentration-time profile in the HFS-MAC.

Supplementary Figure S2. Ceftriaxone monotherapy versus SOC in five strains in the HFS-MAC.

Supplementary Figure S3. Growth rate model in five strains in the HFS-MAC.

**Supplementary Tables**

Supplementary Table S1. Inhibitory sigmoid E_max_ parameters for each sampling day

Supplementary Table S2. MICs in 5 MAC strains used in HFS-MAC versus SOC

**Online methods**

**Supplementary Figure S1. Avibactam concentration-time profile in the HFS-MAC.**

The concentration-time profiles of avibactam achieved extracellularly and intracellularly in each of the six HFS-MAC units that received avibactam. **(A)** shows that avibactam’s extracellular concentrations remained constant at about 15 mg/L within the dosing interval consistent with the study design described in the methods section. The figure with corresponding ceftriaxone concentration-time profiles is shown in the main text. **(B)** shows that the intracellular concentrations were higher and that the intracellular pharmacokinetics differed from the extracellular

**Supplementary Figure S2. Ceftriaxone monotherapy versus SOC in five strains in the HFS-MAC.**

Replicates represent each individual clinical strain and results are shown for mean and standard deviation (error bar). For cfu/mL readouts shown, they are a mean of the direct colony counts on agar and cfu/mL translated from TTP readout from all replicates. **(A)** The concentration-time profiles for 10 replicates per time point for ceftriaxone, azithromycin, ethambutol and rifabutin, achieved in the HFS-MAC, for all five isolates. These are equivalent to intrapulmonary pharmacokinetics. The between HFS-MAC unit variability was less than 10% at all time points for all drugs, satisfying quality control criteria. **(B)** Ceftriaxone monotherapy versus three-drug SOC in strain 1 demonstrated that pattern of microbial kill was the same between SOC and ceftriaxone. Ceftriaxone maximal kill was 1.02 log_10_ cfu/mL below day 0. **(C)** Ceftriaxone monotherapy versus SOC in strain 2 demonstrated that microbial kill was equivalent in the two regimens, but maximal kill was 1.94 log_10_ cfu/mL below day 0. **(D)** In strain 3, the kill rate of ceftriaxone monotherapy was faster than SOC and acheived maximal kill of 2.99 log_10_ cfu/mL below day 0. **(E)** In strain 4, ceftriaxone monotherapy was better than the SOC which failed therapy. Maximal kill of ceftriaxone monotherapy was 3.82 log_10_ cfu/mL below day 0. **(F)** In strain 5, the SOC failed as well while ceftriaxone was highly effective. Maximal kill of ceftriaxone monotherapy was 2.82 log_10_ cfu/mL below day 0.

**Supplementary Figure S3. Growth rate model in five strains in the HFS-MAC.**


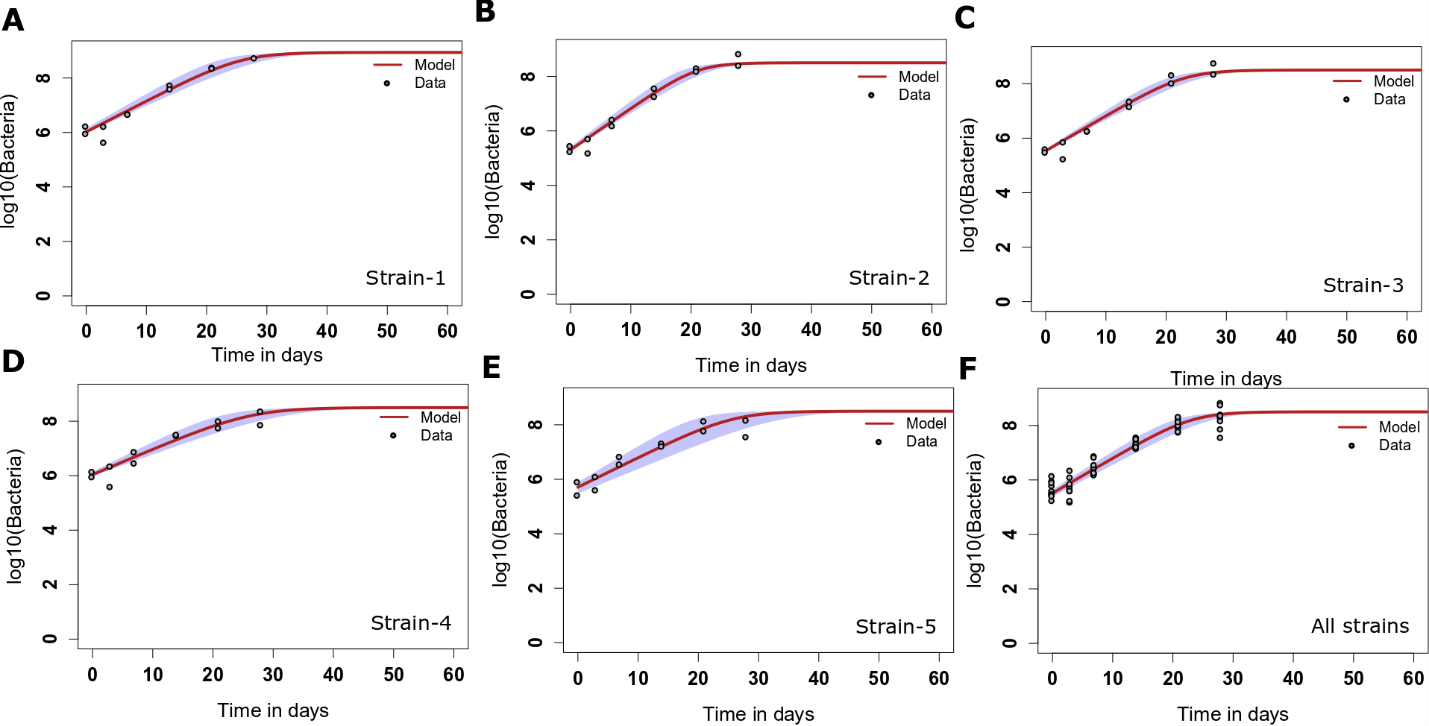


Panels A-E show model fitting to the different MAC strains. The solid line represents the model, the dots represent the HFS generate data and the shaded region represents the 95% CIs. Panel F, is model fitting to all the 5 MAC strains and the 95CIs uncertainty in mode to data fitting.

**Supplementary Table S1. Inhibitory sigmoid E_max_ parameters for each sampling day**

| **Day 7** |  |  |  |  |
| --- | --- | --- | --- | --- |
| Ceftriaxone | 7.38 | 3.56 | 38.92 |  |
| Ceftriaxone + avibactam | 7.41 | 3.14 | 44.65 | 0.802 |
| **Day 14** |  |  |  |  |
| Ceftriaxone | 9.08 | 7.51 | 63.02 |  |
| Ceftriaxone + avibactam | 9.30 | 6.04 | 72.38 | >0.991 |
| **Day 21** |  |  |  |  |
| Ceftriaxone | 9.24 | 6.49 | 97.33 |  |
| Ceftriaxone + avibactam | 10.2 | 5.93 | 100 | 0.917 |
| **Day 28** |  |  |  |  |
| Ceftriaxone | 7.603 | 6.13 | 100 |  |
| Ceftriaxone + avibactam | 7.99 | 5.06 | 100 | 0.973 |

**Supplementary Table S2. MICs in 5 MAC strains used in HFS-MAC versus SOC**

|  | **Strain 1** | **Strain 2** | **Strain 3** | **Strain 4** | **Strain 5** |
| --- | --- | --- | --- | --- | --- |
| Ceftriaxone (mg/L) | 2 | 2 | 0.5 | 2 | 4 |
| Clarithromycin (mg/L) | 0.125 | 0.125 | 0.125 | 1 | 0.5 |
| Ethambutol (mg/L) | 4 | 4 | 0.125 | 4 | 16 |
| Rifabutin (mg/L) | 0.125 | 0.125 | 0.125 | 2 | 0.125 |

**ONLINE METHODS**

**Minimum inhibitory concentration (MIC)**

We used the standard broth microdilution in cation-adjusted Mueller-Hinton broth (CAMHB) to determine the ceftriaxone MIC of MAC [49]. The bacteria were grown to log-phase growth in CAMHB, after which turbidity was adjusted to McFarland standard of 0.5. The turbidity-adjusted cultures were diluted 100-fold in CAMHB to achieve a bacterial density of ~10^5^ CFU/mL. Next, 180 μL of the culture was added to each well of 96-well plates, pre-filled with 20 μL of the drugs at 10x concentration (10-fold dilution). The final drug concentrations ranged between 0.125 mg/L to 128 mg/L in a 2-fold dilution series. Non-treated cultures were used as growth controls. The cultures were incubated at 37°C for seven days in sealed plastic bags. On day 7, the 96-well plates were inspected using an inverted mirror, and MIC was defined as the lowest drug concentration that completely inhibited visible microbial growth in the wells [50]. The experiments were performed twice, with three replicates for each drug concentration.

**Ceftriaxone intracellular static concentration-response study**

THP-1 monocytes were cultured in RPMI-1640 with 10% FBS were activated to macrophages using phorbol myristate acetate (PMA) (10^-9^M final concentration) in 12-well tissue culture plates for 72hr at a cell density of 1.5x10^6^ cells/mL. The adherent macrophages were infected with MAC ATCC#700898 at a bacterium-to-macrophage ratio of 1:1 overnight, after which they were washed twice with warm RPMI-1640. Ceftriaxone in RPMI was added to make final concentrations of 0, 0.125, 0.25, 0.5, 1,2,4,8, and 18 mg/L, and incubated for seven days, after which they were washed twice and lysed with phosphate-buffered saline plus 0.025% Tween-20 (PBS-T). The cultures were then serially diluted, and cultured on Middlebrook 7H9 agar supplemented with 10% oleic acid-catalase-dextrose-albumin (OADC) (herein termed "agar"). Cultures were incubated at 37**°**C for 14 days and cfus counted. In parallel a similar experiment in which 15 mg/L of avibactam was added was performed, *ceteris paribus*. The experiment was performed twice with two replicates per drug concentration. Next, the same experiments were performed in CAMHB, but this time in extracellular MAC, without THP-1 cells.

**Ceftriaxone exposure-effect study in the HFS-MAC**

We performed a ceftriaxone exposure-response study in the HFS-MAC, at a ceftriaxone 8hr half-life [14, 19, 30, 31]. Since %T_MIC_ is associated with the efficacy of cephalosporins, the drug dose selection was to achieve the %T_MIC_ ranging from 0% (nontreated control) to 100%. Briefly, non-activated THP-1 monocytes were infected with MAC ATCC#700898 overnight at an intended MOI of 1:1, followed by inoculation of 20mL of MAC-infected THP-1 cells (density ~1.5*10^5^ cell/mL) into the peripheral compartment of each of eight HFS-MAC units, including the two nontreated controls. Ceftriaxone was administered once daily for 28 days via computerized syringe pumps into the central compartment of HFS-MAC units at seven different exposures, including zero. In addition, a similar number of HFS-MAC were also treated with avibactam at 15 mg/L added to circulating media, except for the highest and lowest ceftriaxone doses. Since we wanted to test the null hypothesis that avibactam does not add to effect of ceftriaxone we did not create a PK system for avibactam, but used a continuous infusion of constant concentration of 15 mg/L (based on peak concentration in ceftazidime/avibactam) as opposed to the β-lactamase inhibitor half-life of 2.7hrs: hypothesis test *in extremes*.

**Ceftriaxone concentration assay**

Ceftriaxone concentrations from the HFS samples were analyzed by liquid chromatography with tandem mass spectrometry (LC-MS). The assay is described in detail in the accompanying online methods. Tazobactam (internal standard, IS), was purchased from Santa Cruz Biotechnology (Santa Cruz, CA, USA), and ceftazidime-d5 (IS) was purchased from Toronto Research Chemicals (Toronto, Canada), respectively. Stock solutions of the standard and IS were prepared in 80:20 methanol:water at a concentration of 1 mg/mL. Calibration curve [ range: 0.005-40 microgram/mL(avibactam) and 0.2-200 microgram/mL (ceftriaxone)], low- and high-quality control samples (LQC and HQC) were prepared by diluting the stock solution in blank medium. For avibactam, solvents for UPLC were: (A) 0.1% aqueous formic acid, and (B) 0.1% formic acid in methanol. For ceftriaxone, solvents were 30:70 10 mM ammonium acetate buffer (pH 6.8): acetonitrile. Samples were diluted 1:20 with IS solution. The transitions used were *m/z* 264 to 96 (avibactam), *m/z* 299 to 138 (tazobactam, IS), *m/z* 555 to 396 (ceftriaxone), and *m/z* 552 to 468 (ceftazidime-d5, IS). The lower limit of quantitation for avibactam was 0.05 μg/ml (extracellular) and 0.025 μg/ml (intracellular). The between day percentage coefficient of variation (%CV) for analysis of low and high (brackets) quality controls for avibactam extracellularly, and intracellularly were 7% (2%), and 3% (2%), respectively. The intraday %CV for avibactam extracellularly, and intracellularly were 5% (2%), and 5% (3%), respectively. The lower limit of quantitation for ceftriaxone was 0.2 μg/ml (extracellular) and 0.1 μg/ml (intracellular). The between day %CV for ceftriaxone extracellularly, and intracellularly were 6% (2%), and 6% (3%), respectively. The intraday %CV for ceftriaxone extracellularly, and intracellularly were 7% (4%), and 8% (4%), respectively. Avibactam was detected using negative ESI while ceftriaxone was detected using positive ESI in MRM mode.

**Ceftriaxone clinical dose selection *in silico* experiments**

We examined 1G, 2G, and 3G once daily doses in MCE simulations, administered intravenously over 30 minutes. The following ceftriaxone one-compartment model PK parameter estimates (between-individual variability as a % coefficient of variation) from Fraschnini et al. were entered into the domain of input of sub-routine PRIOR in ADAPT: total clearance 1.29 L/hr (9%) and volume of 12.91 L (17.43%) [19]. External model validation was performed by comparing the clearances, the volume of distribution, the peak, and trough concentrations identified after the 2G intravenous dose in a separate study by Pollock et al [37]. The probability of target attainment (PTA) was calculated using the MIC distribution of our 59 clinical strains.
